# Massive GI bleeding in a patient with 2 small AVMs in the small intestine: a case report

**DOI:** 10.1186/1757-1626-3-39

**Published:** 2010-01-28

**Authors:** Tatiana B Jacobson, Victor O Kolade

**Affiliations:** 1 Department of Internal Medicine, University of Tennessee, Chattanooga, TN, USA

## Abstract

A 53 year-old Caucasian man with no previous history of gastrointestinal bleeding presented with sudden, massive hematochezia and abdominal pain; his hemoglobin dropped from 12 to 8.3. Colonoscopy revealed coagulated blood in a diverticulum, but bleeding recurred after cautery of the lesion.

Repeated upper and lower gastrointestinal (GI) endoscopy, visceral selective angiogram, bleeding scan, and Meckel diverticulum scan did not locate the source of bleeding. Further investigation with capsule endoscopy demonstrated two arteriovenous malformations in the small bowel.

Wireless capsule endoscopy is a sensitive and specific test for overt obscure gastrointestinal bleeding. Clinicians need not hesitate to employ this procedure when other diagnostic modalities fail.

## Case

A 53 year-old Caucasian man with a history of gastroesophageal reflux disease but no previous history of gastrointestinal bleeding and an unremarkable family history presented to the Emergency Room with sudden-onset, massive hematochezia of 19 hours duration and mild right lower quadrant abdominal pain. He developed an acute drop in hemoglobin from 12.0 g/dL to 8.3 g/dL within 9 hours of admission. His platelet count, coagulation studies, liver and renal function tests were normal. Emergent esophagogastroduodenoscopy, colonoscopy and push enteroscopy were performed; coagulated blood was observed at the site of a nipple of one colonic diverticulum. Epinephrine injection and cautery anticoagulation were done at that site, but the patient continued to have massive hematochezia followed by maroon stool. Extensive evaluation including an abdominal contrast computed tomography (CT) scan, a repeat upper and lower gastrointestinal (GI) endoscopy, visceral selective angiogram, bleeding scan, and Meckel diverticulum scan failed to identify the source of bleeding. He required transfusion of 15 units of packed red blood cells for correction of anemia.

**Figure 1:**
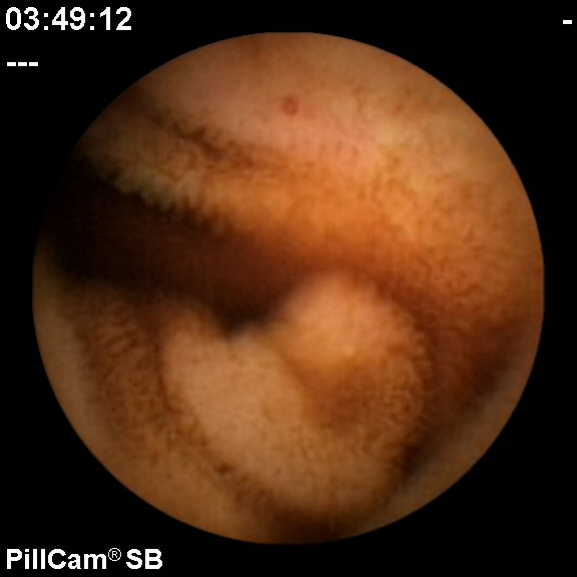
**Capsule endoscopy image showing an arteriovenous malformation in the small bowel at approximately 12 o'clock**.

Further investigation with capsule endoscopy demonstrated two small arteriovenous malformations (AVMs) in the small bowel (see Figure 1). The bleeding stopped and did not recur. The patient was discharged from the hospital in stable condition. He did not have any gastrointestinal complaints or recurrence of bleeding at outpatient follow-up one month after hospital discharge.

## Discussion

Obscure gastrointestinal bleeding (OGIB) refers to intermittent or continuous loss of blood in which the source has not been identified after upper endoscopy and colonoscopy [1]. It may be occult bleeding, detected by fecal occult tests or alluded to by unexplained iron deficiency anemia, or overt bleeding such as our patient experienced. The diagnostic evaluation can include repeat upper endoscopy, colonoscopy, push enteroscopy, radionuclide bleeding scan, angiography, and exploratory laparotomy with intraoperative enteroscopy [2].

AVMs of the small intestine can be described as abnormally dilated submucosal veins. The pathogenesis is uncertain. AVMs appear to occur equally in men and women, and there is no predilection for race. Bleeding may be either occult or severe. The most common presentation is with hematemesis or melena. Although massive upper GI bleeding with hemodynamic compromise may occur, fatal upper GI hemorrhage from AVMs is extremely rare.

Wireless capsule endoscopy (CE) has become the procedure of choice for the evaluation for obscure GI bleeding, with a diagnostic yield of 49-63% [3-5]. AVMs accounted for up to 44% of these diagnoses [5]. The diagnostic yield of CE was highest - 92.3% - in patients with ongoing obscure overt bleeding such as ours [4]. CE has a higher yield for vascular and all findings in OGIB as compared to push enteroscopy [3]; its sensitivity is 89%, specificity 95%, positive predictive value 97%, and negative predictive value, 83% [4]. It is thus the gold standard for diagnosis of OGIB. Abdominal computed tomography is appropriate to exclude intestinal obstruction or stenosis before CE is performed.

Double - balloon enteroscopy can be used to treat lesions identified by CE, or as a second-line test when CE is negative and high suspicion of small bowel pathology persists [6].

In our case, capsule endoscopy revealed two small AVMs as a cause of significant obscure GI bleeding leading to profound anemia requiring a blood transfusion; other studies, including repeated upper and lower endoscopy, visceral selective angiogram, bleeding scan, and Meckel scan were unrevealing.

An AVM is a rare cause of obscure GI bleeding which can be a diagnostic challenge for clinicians. It can cause profound anemia and thus requires prompt evaluation. CE has been shown to demonstrate a cause of GI bleeding localized to the small intestine when other modalities failed to do so.

## Abbreviations

CT: computed tomography; GI: gastrointestinal; AVM: arteriovenous malformation; OGIB: obscure gastrointestinal bleeding; CE: capsule endoscopy.

## Consent

The subject of this case report is now deceased; none of his relatives were available to provide written informed consent.

## Competing interests

The authors declare that they have no competing interests.

## Authors' contributions

TBJ analyzed and interpreted the patient data regarding the arteriovenous malformation; she initiated the literature review. VOK revised the literature review and was a major contributor in writing the manuscript. All authors read and approved the final manuscript.
